# Adult-onset CblC deficiency: a challenging diagnosis involving different adult clinical specialists

**DOI:** 10.1186/s13023-022-02179-y

**Published:** 2022-02-02

**Authors:** Silvia Kalantari, Brigida Brezzi, Valeria Bracciamà, Antonella Barreca, Paolo Nozza, Tiziana Vaisitti, Antonio Amoroso, Silvia Deaglio, Marco Manganaro, Francesco Porta, Marco Spada

**Affiliations:** 1 Department of Medical Sciences, University of Turin, Turin, Italy; 2 Nephrology and Dialysis Unit, Azienda Ospedaliera “SS. Antonio e Biagio e Cesare Arrigo”, Alessandria, Italy; 3 Anatomia e Istologia Patologica, Città della Salute e della Scienza University Hospital, Turin, Italy; 4 S.C. Anatomia e Istologia Patologica, Azienda Ospedaliera “SS. Antonio e Biagio e Cesare Arrigo”, Alessandria, Italy; 5 Immunogenetics and Biology of Transplantation, Città della Salute e della Scienza University Hospital, Turin, Italy; 6 Department of Pediatrics, Città della Salute e della Scienza University Hospital, University of Torino, Piazza Polonia 94, 10126 Turin, Italy

**Keywords:** Methylmalonic aciduria and homocystinuria, Cobalamin C deficiency, CblC, Adult-onset, Homocysteine, Neuropsychiatric presentation, Renal function decline, aHUS

## Abstract

**Background:**

Methylmalonic aciduria and homocystinuria, CblC type (OMIM #277400) is the most common disorder of cobalamin intracellular metabolism, an autosomal recessive disease, whose biochemical hallmarks are hyperhomocysteinemia, methylmalonic aciduria and low plasma methionine. Despite being a well-recognized disease for pediatricians, there is scarce awareness of its adult presentation. A thorough analysis and discussion of cobalamin C defect presentation in adult patients has never been extensively performed. This article reviews the published data and adds a new case of the latest onset of symptoms ever described for the disease.

**Results:**

We present the emblematic case of a 45-year-old male, describing the diagnostic odyssey he ventured through to get to the appropriate treatment and molecular diagnosis. Furthermore, available clinical, biochemical and molecular data from 22 reports on cases and case series were collected, resulting in 45 adult-onset CblC cases, including our own. We describe the onset of the disease in adulthood, encompassing neurological, psychiatric, renal, ophthalmic and thromboembolic symptoms. In all cases treatment with intramuscular hydroxycobalamin was effective in reversing symptoms. From a molecular point of view adult patients are usually compound heterozygous carriers of a truncating and a non-truncating variant in the *MMACHC* gene.

**Conclusion:**

Adult onset CblC disease is a rare disorder whose diagnosis can be delayed due to poor awareness regarding its presenting insidious symptoms and biochemical hallmarks. To avoid misdiagnosis, we suggest that adult onset CblC deficiency is acknowledged as a separate entity from pediatric late onset cases, and that the disease is considered in the differential diagnosis in adult patients with atypical hemolytic uremic syndromes and/or slow unexplained decline in renal function and/or idiopathic neuropathies, spinal cord degenerations, ataxias and/or recurrent thrombosis and/or visual field defects, maculopathy and optic disc atrophy. Plasma homocysteine measurement should be the first line for differential diagnosis when the disease is suspected. To further aid diagnosis, it is important that genes belonging to the intracellular cobalamin pathway are included within gene panels routinely tested for atypical hemolytic uremic syndrome and chronic kidney disorders.

**Supplementary Information:**

The online version contains supplementary material available at 10.1186/s13023-022-02179-y.

## Background

Vitamin B12 deficiency is a well-defined common clinical entity in adult medicine. Autoimmune atrophic gastritis is the most common cause, besides deficient nutritional intake, and the study of its pathophysiology roots back to the XIX century [1]. The combination of atrophic gastritis, macrocytic anemia, atrophic glossitis and accompanying neurological signs (i.e. numbness and ataxia) due to demyelination of posterior and lateral columns of the spinal cord are easily recognized and treated in adult patients nowadays.

Less widely known are rare inherited disorders of vitamin B12 metabolism, either leading to deficiency of the vitamin itself or of its functions due to impaired intracellular transformation into adenosyl and methylcobalamin, its actual active metabolic forms. All the steps of vitamin B12 absorption and intracellular metabolism have been reviewed elsewhere [2]. The focus of the present review is methylmalonic aciduria and homocystinuria, CblC type (OMIM #277400), the most common among inherited disorder of cobalamin intracellular metabolism, first described more than five decades ago [3].

**Fig. 1:**
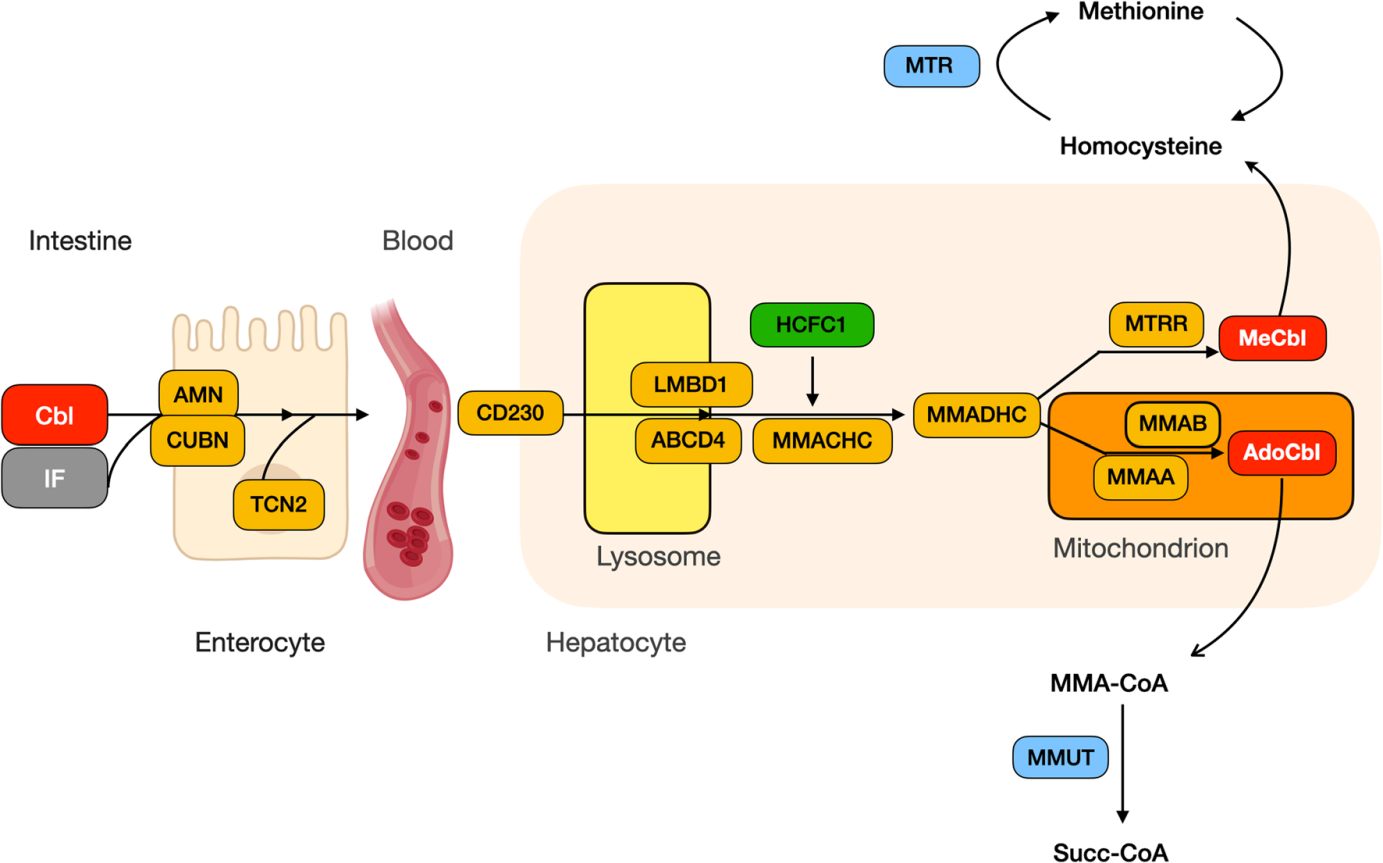
Schematic representation of cobalamin metabolism. Dietary vitamin B12 (Cbl) is bound by intrinsic factor (IF) in the stomach, the vitamin B12-instrinsic factor complex is absorbed by enterocytes in the ileum through the cubam receptor, formed by cubilin (CUBN) and amnionless (AMN). Cobalamin is then transferred onto transcobalamin 2 (TCN2) and transported in the bloodstream. The transcobalamin 2-cobalamin complex is taken up by hepatocytes through the TC2 receptor (CD230) and transferred to lysosomes, from which it is released by the membrane-bound transport proteins LMBD1 and ABCD4 and processed by MMACHC (whose transcription is controlled by HCFC1). MMADHC binds to MMACHC and then processed cobalamin is either directed towards Methylcobalamin (MeCbl) synthesis through methionine synthase reductase (MTRR) or to the mitochondrion, where Adenosylcobalamin (AdoCbl) is synthetized thanks to MMAA and MMAB proteins. MeCbl is a cofactor for the enzyme methionine synthase (MTR), involved in remethylation from homocysteine to methionine, while AdoCbl is a cofactor of methylmalonyl-CoA mutase (MMUT), which catalyzes the transformation of L-Methylmalonyl-CoA (MMA-CoA) into Succynil-CoA, which can then be used in the Krebs cycle [2]

Despite being a rare disorder, newborn screening revealed an incidence of CblC deficiency higher [1:100,000 in New York City [4] and 1:121,622 in New Jersey [5]] than previously expected [1:200,000 [6]]. The disorder is caused by MMACHC impairment, most commonly due to pathogenic variants affecting *MMACHC* gene, less commonly due to variants affecting the neighboring gene *PRDX1*, causing *MMACHC* silencing [7]. Due to defective *MMACHC* gene product, methylcobalamin and adenosylcobalamin cannot be produced within the cell. They are essential cofactors for correct remethylation of homocysteine to methionine and for the conversion of methylmalonic into succinic acid, respectively (Fig. 1). Their deficiency causes elevated total plasma homocysteine (Hcy), plasma and urine methylmalonic acid (MMA) and low methionine (Met) levels, all three being typical hallmarks of the disease. Folate and vitamin B12 in plasma are instead normal. Because of its onset of symptoms the disorder has been historically classified as early-onset CblC disease, where the first presentation of disease is within the first year of life [4 years of age according to some authors [8]], and late-onset CblC disease, where the first symptoms appear later on [9]. About 90% of patients which have been reported in the medical literature had an early onset of the disease [9]. The late onset form is much rarer, with no more than 150 cases reported up to now [8–11]. The disease is thus considered to be mostly pediatric and there is a scarce knowledge of its presentation in adults.

Early-onset disease is characterized by feeding difficulties, failure to thrive, hypotonia, developmental delay, acute encephalopathy, seizures, metabolic acidosis, hydrocephalus, atypical hemolytic uremic syndrome (aHUS), glomerulopathy, chronic renal failure and pulmonary artery hypertension. Visual impairment due to pigmentary retinopathy, nystagmus and optic atrophy are also frequently observed. In some infants, the disease is already apparent prenatally with heart anomalies and intrauterine growth restriction. Anemia, neutropenia and pancytopenia are quite common [2]. Neonatal screening has allowed prompt identification of the disease by detection of elevated propionylcarnitine (C3) and low methionine on mass spectrometry analysis of dried blood spots for over a decade in most developed countries [5]. Second-tier testing with MMA and Hcy allows then to reach final diagnosis. Despite early identification and treatment, prognosis of the early onset form is still poor, with neurological and visual symptoms often worsening over time [5]. HUS and pulmonary hypertension are often the main presenting symptoms in preschool children with late onset CblC. Neuropsychiatric symptoms are instead more likely in adolescent patients [6]. Contrary to early onset cases, prompt initiation of therapy in late onset cases gives immediate biochemical and clinical results, underlining the importance of the diagnosis [9].

The adult-onset form of disease was described for the first time in 2001 [12] and very few cases have been published ever since. Despite being classified together with pediatric late onset cases, they have some unique characteristics that would account for their evaluation as a separate entity. A thorough analysis and discussion of cobalamin C defect presentation in adult patients had never been extensively done and is the purpose of this review. We have collected the available clinical, biochemical and molecular data and we here describe the insidious and easily misdiagnosed onset of the disease in adulthood. We furthermore present the emblematic case of 45-year-old male with the latest onset of disease ever recorded and describe the diagnostic odyssey he ventured through to get to a diagnosis. Our ultimate purpose is to raise awareness for the adult onset of this rare, but treatable disease.

## Case report

**Fig. 2:**
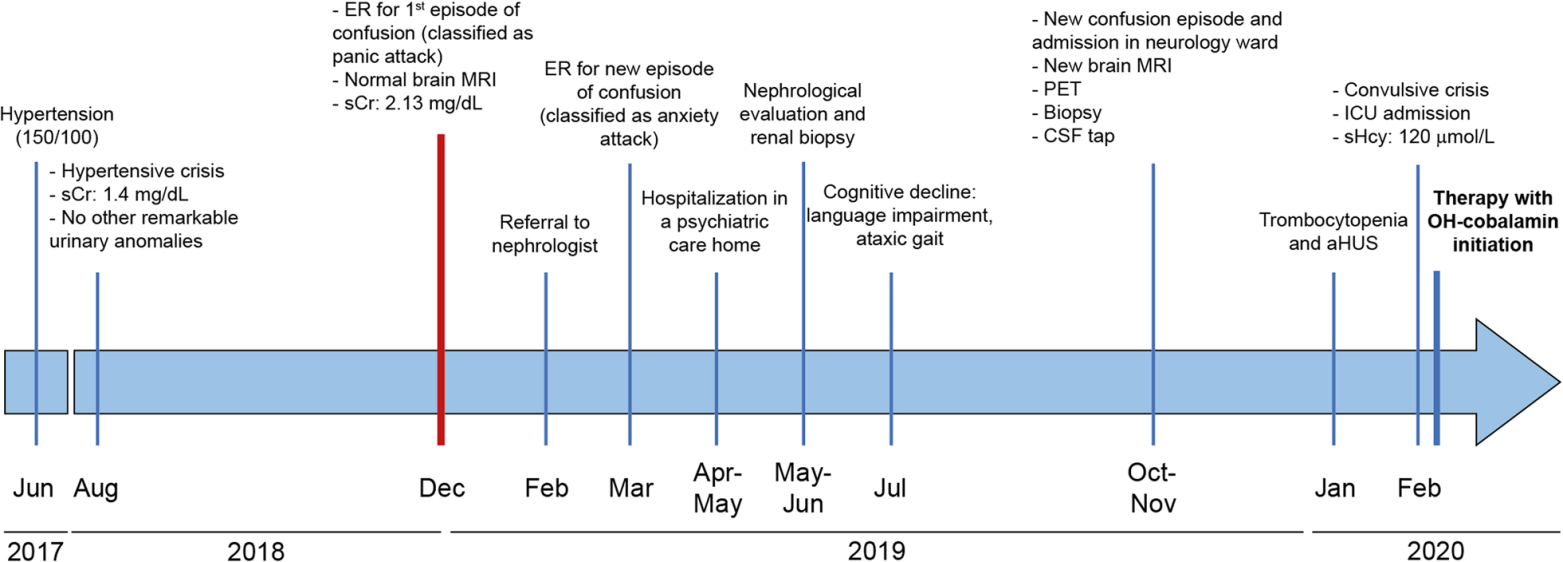
Case report storyline. Timeline of clinical events from the onset of arterial hypertension to the diagnosis of methylmalonic aciduria and homocystinuria, CblC type of the reported case of adult-onset CblC disease. ER, Emergency Room; aHUS, atypical Hemolytic Uremic Syndrome, OH-cobalamin, Hydroxycobalamin

A 45-year-old Italian male business manager came to clinical attention due to psychiatric symptoms, cognitive decline and slowly worsening renal function (Fig. 2). Previously to these symptoms, he only had an isolated acute pericarditis when he was 28 and was followed for diastolic hypertension from 43 years of age (average pressure 140/90).

At age 45 he had several episodes of confusion, during which he would not be able to interact with the surrounding environment and would feel slowed down. These episodes were classified as panic attacks due to anxiety in the context of a depressive disorder. Moreover, the patient reported insomnia, visual hallucinations, sight impairment and difficulty in following complex reasoning in the previous months. A brain CT scan and MRI with contrast medium did not show any significant organic lesion, nor did the ophthalmologic visit. Psychiatric follow-up was suggested. Due to worsening depressive symptoms, he was hospitalized in a psychiatric care home for two months where a decline in memory and attention capability were noted. All the symptoms were ascribed to stress and the patient inability to cope with the work-place demands as a manager.

**Fig. 3:**
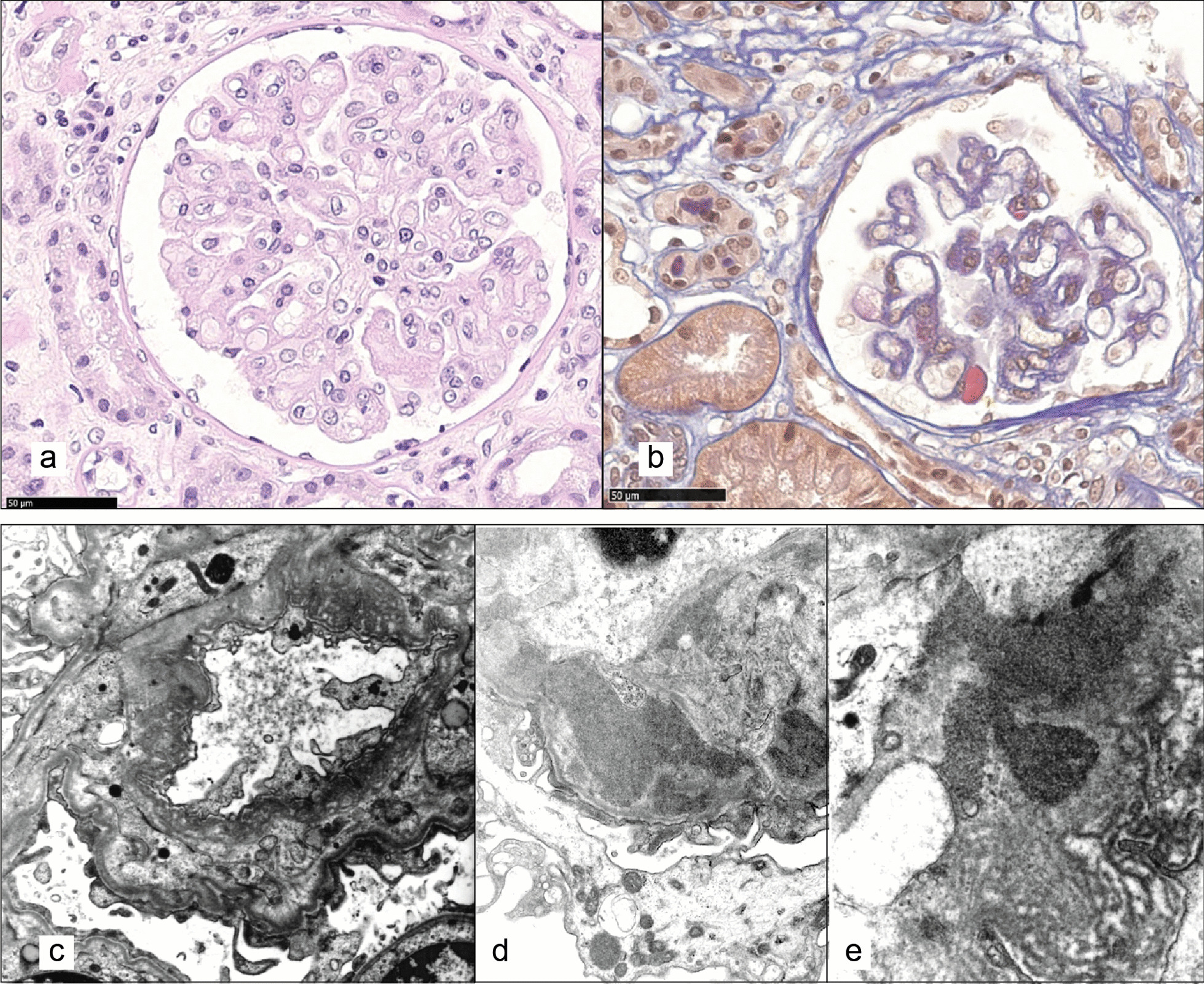
Renal biopsy. Optical microscopy (**a**, **b**) and electron microscopy (**c**–**e**) of the reported case of adult-onset CblC disease. **a** The glomerular basement membranes are diffusely slightly thickened and show extensive double contours or collapse (PAS, original magnification × 400). **b** pseudothrombi are occasionally observed in glomerular capillary lumens (AFOG, original magnification × 400). **c** the glomerulus shows extensive duplication of basement membranes (“multilayering” aspect) associated with subendothelial expansion, endothelial swelling and loss of normal fenestration (chronic microangiopathic damage; original magnification × 5200). **d**, **e** Electron dense deposits with a vaguely structured annular or microtubular appearance are evident in the basement membranes (mainly subendothelial and intramembranous) and in the mesangium (original magnification × 15,500 in d and e)

In the same months, a decline in renal function (serum creatinine 2.13 mg/dL after the first confusion episode, reference range 0.4–1.0 mg/dl) brought him to the attention of the nephrology ward. His serum creatinine (sCr) values had been within normal range before age 44 (latest sCr values from 1 year before 1.12 mg/dL). Proteinuria was detected on urine exams (values ranging from 207 to 500 mg/24 h, reference range < 300 mg/24 h). A renal biopsy was asked for after a few months of follow-up, showing chronic thrombotic microangiopathy features (extensive double contours, focal mesangial cell interposition) and early intravascular thrombi in occasional small vessels. However, immunofluorescence demonstrated parietal and mesangial granular polytypic deposits with strong positivity for IgG and C1q and moderate positivity for IgM and C4 associated with the presence of occasional protein pseudothrombi in the capillary lumens and vascular deposits with intense positivity for IgM and C3 and discrete positivity for C1q and C4, in an overall picture suggestive of cryoglobulinemic glomerulonephritis (Fig. 3a, b). Electron microscopy, showing electrodense deposits with vague annular and microtubular structuring, seemed to confirm this diagnostic hypothesis (Fig. 3b–d). Further targeted investigations did not provide other laboratory findings in support neither of cryoglobulinemia (negative Rheumatoid factor test and HCV antibodies, no circulating cryoglobulins and no hypocomplementemia), nor of thrombotic microangiopathy (Platelets 156,000/µL [reference values 156,000–405,000/µL], absent schistocytes on peripheral blood smear, LDH 458 U/L (reference values 230–500 U/L), haptoglobin 93 mg/dL (reference values 40–240 mg/dL), normal levels of ADAMTS13 and complement factor H, no anti-factor H antibodies), while genetic investigation through next-generation sequencing (NGS) and analysis of a panel of genes (*CFH*, *CFI*, *CFB*, *C3*, *CD46*, *THBD*, *DGKE*, *CFHR1*, *CFHR3* and *CFHR5)* causative of complement abnormalities associated with aHUS did not identify pathogenic variants.

**Fig. 4:**
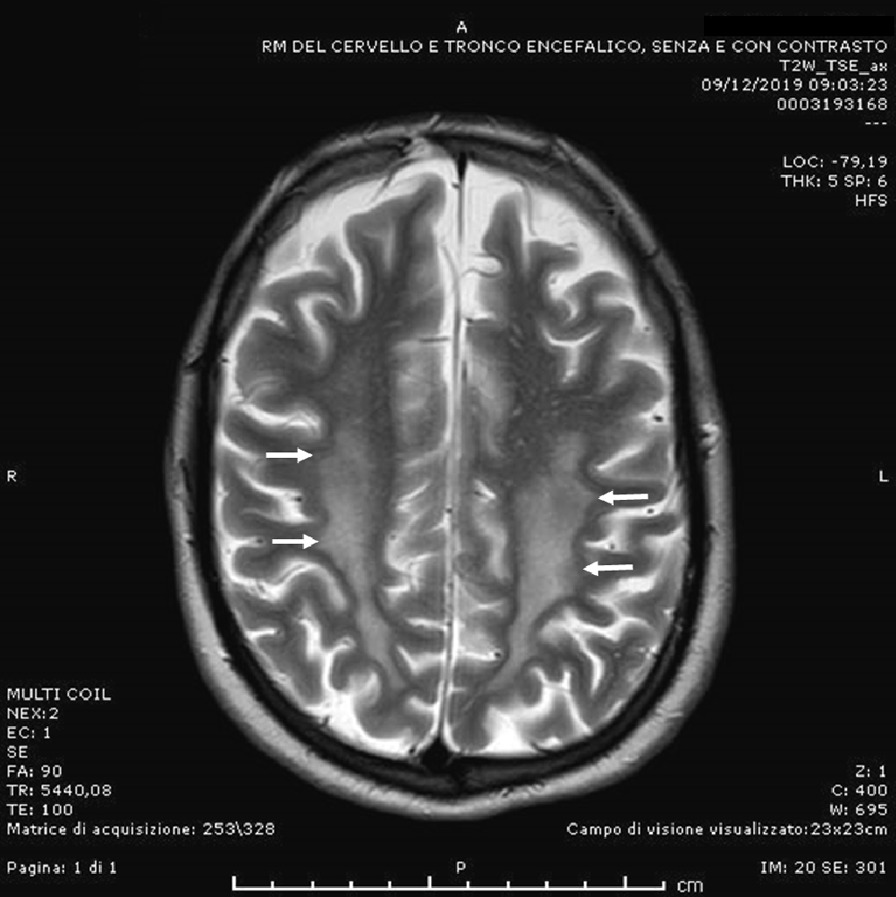
Brain MRI. In the T2-weighted sequences, foci of altered hyperintense signal of the bilateral fronto-temporo-parieto-occipital subcortical white matter are evident (white arrows)

**Fig. 5:**
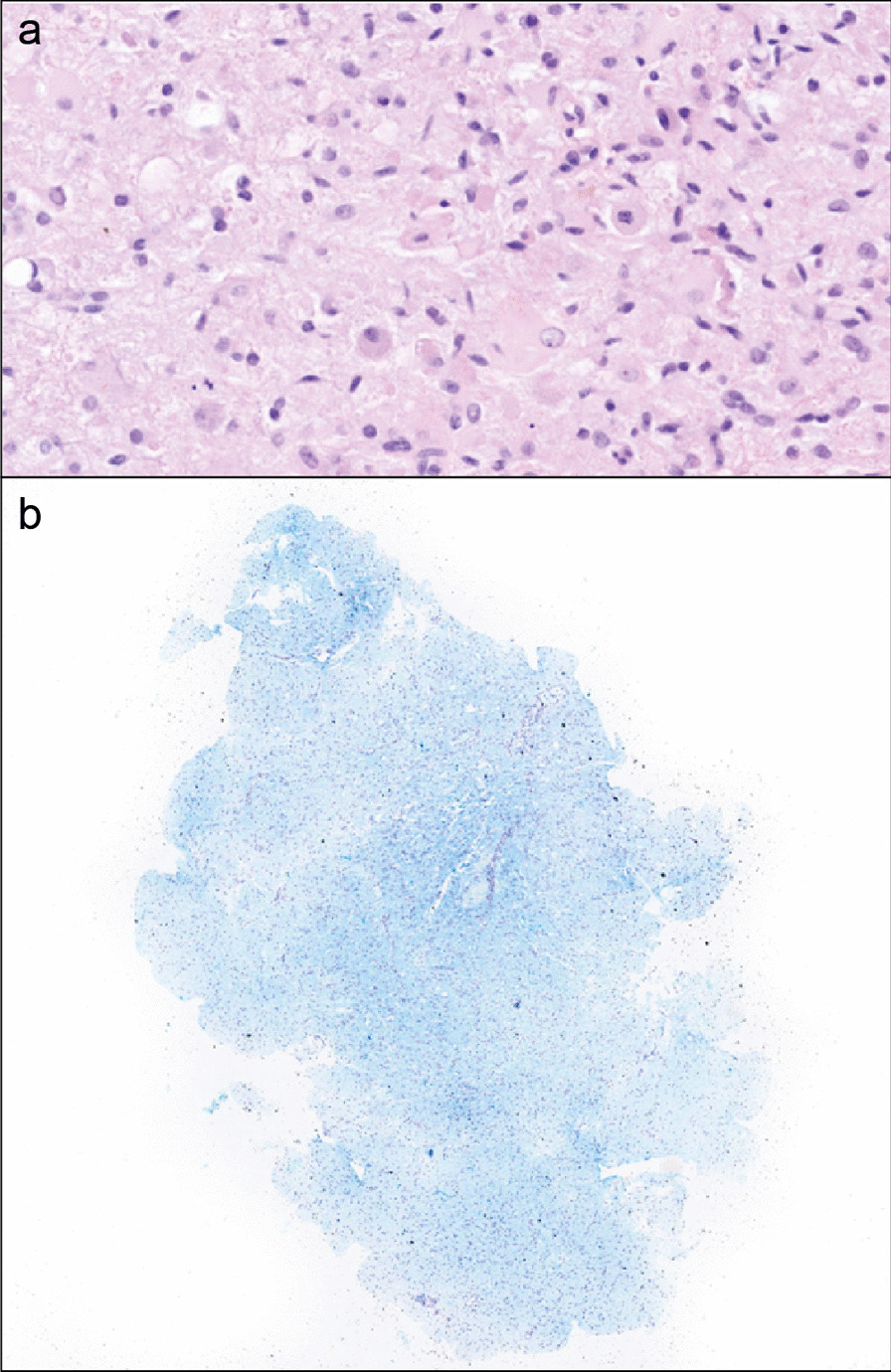
Brain biopsy. Hematoxylin–eosin image shows an increase in the cell density of the white matter and the presence of macrophages (**a**) and Luxol fast blue staining highlighting the presence of foci of demyelination (**b**)

Over time the patient withdrew from social life and was unable to continue working. He had a severe cognitive decline with psychomotor slowing, language impairment, and a spastic ataxic gait. He was thus referred to the neurology ward, where exaggerated deep tendon reflexes in all extremities, an abnormal Babinsky sign and a bilateral positive Hoffmann’s reflex were observed. A new brain MRI revealed extensive hyperintense T2 signal in subcortical white matter in bilateral temporal, occipital and parietal areas, similar to those found in posterior reversible encephalopathy syndrome (PRES; Fig. 4). Cortical atrophy signs were also apparent. Examination of the fundus oculi did not reveal any abnormality (normal optic nerve and macula). A brain PET was suggestive of an inflammatory process in the occipital cortex bilaterally. Cerebrospinal fluid analysis and screening for autoimmune CNS diseases were negative. Due to the uncertain nature of the lesions and the further worsening in MRI imaging, a brain biopsy was performed, which showed a hypercellularity of the white matter due to the presence of macrophages and, to a lesser extent, small lymphocytes, the latter in perivascular distribution. Foci of demyelination were seen, while no signs of neoplasms, vasculitis or encephalitis were present (Fig. 5). Treatment with high doses corticosteroids was initiated, without any clinical response from the patient, who was discharged with a strict follow-up program.

Two weeks later, he was admitted to the hospital for an episode of aHUS with thrombocytopenia (87,000/µL), peripheral schistocytes (> 1%), LDH increase (557 U/L) and C3 decrease (77 mg/dL, reference values 84–160 mg/dl), together with increased blood pressure. His hemoglobin was 12.6 g/dl, MCV was 94 µmm^3^ (reference values 80–100 µmm^3^) and serum vitamin B12 was 875 pg/ml (reference values 212–911 pg/ml). He was treated with eculizumab, without improvement. The lack of response to eculizumab suggested the possibility of a metabolic disorder: as a consequence, homocysteine plasma values were measured and resulted in severely increased levels (> 130 μmol/L). In the meantime, the patient was transferred to the intensive care unit due to repeated seizures, the subsequent onset of acute respiratory distress syndrome, requiring intubation and artificial ventilation, and by further deterioration of renal function, prompting continuous renal replacement therapy initiation. On the basis of the severe hyperhomocysteinemia, in agreement with metabolic disease experts, further targeted investigations were carried out revealing a methylmalonic acidemia with homocystinuria (plasma methionine 6.5 μmol/L, plasma MMA 119 μmol/L, urinary MMA 310.5 μmol/mmol), allowing a clinical diagnosis of cobalamin C deficiency. Therapy with parenteral hydroxycobalamin was promptly initiated. Following therapy initiation, the patient had a marked improved of neurological symptoms, partial improvement of brain imaging features, partial improvement of glomerular filtration rate (sCr 3.3 mg/dL). Weaning from hemodialysis and normalization of biochemical parameters (Hcy 20 μmol/L) were possible within a few weeks. Therapy at discharge was 5 mg intramuscular (im) OH-cobalamin 5 days/week, betaine 9 g/day and 5 mg/3 days a week folic acid.

Genetic testing through NGS sequencing of a panel of genes associated to intracellular cobalamin disorders identified two heterozygous variants in *MMACHC* gene: c.220delA (p.Met74Cysfs*2) and c.395_397delGAC (p.Arg132del). Neither has been published in the medical literature before, but in silico predictions allowed to classify the former as likely pathogenic (Class 4) and the latter as uncertain significance (VUS, Class 3). A family segregation study was possible only for the mother of the patient, as the father had died and the patient had no siblings nor children. The investigation showed the maternal origin of the c.220delA (p.Met74Cysfs*2) variant and confirmed that the two identified variants in the patient were in compound heterozygosity.

At the time of writing, the patient had a complete neurocognitive improvement, and was able to resume his usual work activity. One year later, while he continues maintenance therapy with hydroxocobalamin 5 mg im 5 days/week, betaine 6 g/day and folic acid 5 mg 3 days/week, brain MRI shows a clear reduction in areas of impaired white matter signaling, signs of hemolysis are absent, platelets are persistently normal, creatinine is settled at 2.0 mg/dL and blood pressure is well controlled with antihypertensive therapy.

### Adult-onset CblC patients

We performed a systematic review of the literature searching for adult onset of CblC deficiency. The PubMed database was interrogated using key terms to uniquely identify the disease and time of onset, including “cobalamin; CblC; MMACHC; combined methylmalonic aciduria homocystinuria; MMA; homocysteine” combined with “early onset” and “late onset”. Only review, case report and case series reporting patients with disease onset at 18 years old or older were included. The analysis resulted in 22 manuscripts complying with the inclusion criteria. Data on clinical course, biochemical and molecular parameters were not consistently included in all the reports found.

Overall, in the literature there are 44 adult-onset CblC cases reported, 19 females and 25 males. Data on our own patient, described in the present review for the first time, was also included. The overall results thus encompassed 45 adult-onset CblC case, 19 females and 26 males (Table 1). Among these, two cases (Patient 29 and 30) were asymptomatic and the disease onset corresponded to the age at which the typical biochemical abnormalities of the disease were first noticed. One clinically asymptomatic female (Patient 29) was identified thanks to her child’s newborn screening showing low carnitine [13]. One apparently clinically asymptomatic male (Patient 30) patient was identified after diagnosis of his symptomatic sibling [14].

**Table 1:** Cohort of patients diagnosed with adult-onset of Cobalamin C disease

Number	Age at onset/diagnosis (years)	Gender	Clinical onset	Following signs and symptoms	Plasma Homocysteine at onset (μmol/L)	Genotype		References
						Allele 1	Allele 2	
1	18/18	F	Lower limbs weakness	Neuropathy, psychiatric symptoms, seizures	273.3	c.1A > G p.Met1Val	c.445_446del p.Cys149HisfsTer16	[8]
2	18/19	F	Lower limbs weakness	Psychiatric symptoms, ataxia, cortical atrophy, thoracic cord atrophy, scoliosis	138	c.482G > A .Arg161Gln	c.445_446del p.Cys149HisfsTer16	[32]
3	18/19	F	Neuropsychiatric symptoms	Cognitive decline, neuropathy, psychiatric symptoms, ataxia, hyperintensity of basal ganglia and cerebellum, cervical and thoracic cord atrophy, scoliosis	69.5	c.482G > A p.Arg161Gln	c.445_446del p.Cys149HisfsTer16	[8]
4	18/20	M	Neuropsychiatric symptoms	Neuropathy, psychiatric symptoms, lower limb weakness, cortical atrophy, thoracic cord atrophy, scoliosis	193.4	c.482G > A p.Arg161Gln	c.656_658del p.Lys220ArgfsTer71	[8]
5	18/20	M	HUS	Nephrotic syndrome, hematuria, renal failure	–	c.82-12_82-9delTTTC	c.271dupA p.Arg91LysfsTer14	[18]
6	18/39	F	Pulmonary embolism	Cognitive decline, neuropathy, psychiatric symptoms, myoclonus, dysarthria, dysmetria, adiadochocinesia, tetraparesis, seizures, recurrent thrombosis, glomerulonephritis, leukoencephalopathy, cortical atrophy, corpus callosum agenesis	172	c.365A > G p.His122Arg	c.457C > T p.Arg153Ter	[33]
7	19/19	F	HUS	Acute renal failure, nephrotic syndrome, hematuria, severe neurologic impairment	285	c.566G > A p.Arg189His	c.271dupA p.Arg91LysfsTer14	[31]
8	19/20	F	Psychiatric symptoms, cognitive decline	Anemia, cortical atrophy, thoracic cord atrophy, scoliosis	155	c.452A > G p.His151Ala	c.452A > G p.His151Ala	[32]
9	19/20	F	Neuropsychiatric symptoms, renal disease	Cognitive decline, psychiatric symptoms, renal dysfunction, anemia, cortical atrophy, cervical and thoracic cord atrophy	115.2	c.452A > G p.His151Ala	c.452A > G p.His151Ala	[8]
10	19/20	M	Legs paraplegia	Cognitive decline, neuropathy, bilateral legs paraplegia, deep veins thrombosis	27.9	–	–	[12]
11	19/29	M	Lower limbs weakness, cognitive decline	Ataxia, cortical atrophy, thoracic cord atrophy	114.2	c.482G > A p.Arg161Gln	c.658_660del p.Ala221GlyfsTer7	[8]
12	20/21	M	HUS	Renal failure, malignant hypertension	185	c.389A > G p.Tyr130Cys	c.271dupA p.Arg91LysfsTer14	[17]
13	20/22	F	Psychiatric symptoms	Isolated psychiatric presentation	128	c.365A > G p.His122Arg	c.609G > A p.Trp203Ter	[8]
14	20/22	M	Neurological symptoms	Cognitive decline, pyramidal signs, lower limb weakness, seizures, cortical atrophy, anorexia	230.97	c.482G > A p.Arg161Gln	c.609G > A p.Trp203Ter	[34]
15	20/34	F	Decline in renal function	Proteinuria, microhematuria, anemia	90	c.388 T > C p.Tyr130His	c.666C > A p.Tyr222Ter	[21]
16	22/22	F	Lower limbs weakness, cognitive decline	Increased tendon reflex in upper limbs, decreased tendon reflex in lower limbs, positive Babinski sign, neuropathy	79.8	c.482G > A p.Arg161Gln	c.609G > A p.Trp203Ter	[35]
17	23/23	M	Ataxia	Cognitive decline, numbness and partial paralysis in the lower limbs	167	c.392_394del p.Gln131del	c.392_394del p.Gln131del	[13]
18	23/23	M	Lower limbs weakness	Mild memory impairment, progressive spastic paraplegia, bilateral pyramidal tract signs	93.6	c.482G > A p.Arg161Gln	c.609G > A p.Trp203Ter	[32]
19	24/24	M	Neuropsychiatric symptoms	Cognitive decline, pyramidal signs, hyporeflexia, hyperesthesia, lower limbs weakness, anorexia, coma	100.22	c.482G > A p.Arg161Gln	c.217C > T p.Arg73Ter	[34]
20	24/25	F	Progressive gait disturbance	Myelopathy, lower limb weakness	125	c.347 T > C p.Leu116Pro	c.271dupA p.Arg91LysfsTer14	[36]
21	24/31	F	Neuropsychiatric symptoms	Lower limbs weakness, longitudinally extensive transverse myelitis in cervical spinal cord	75.4	c.463G > C p.Gly155Arg	c.609G > A p.Trp203Ter	[8]
22	25/27	M	Neurological symptoms	Cognitive decline, lower limbs weakness, pyramidal signs, cortical atrophy, coma	111.88	c.482G > A p.Arg161Gln	c.440_441del p.Cys149HisfsTer32	[34]
23	26/26	M	Lower limbs weakness	Progressive spastic paraplegia, pyramidal signs, generalized tonic–clonic seizures, optic nerve atrophy, cortical atrophy	97.7	c.565C > A p.Arg189Ser	c.567dupT p.1190YfsTer13	[32]
24	26/28	F	Maculopathy	Decreased central vision, mild photoaversion	–	c.482G > A p.Arg161Gln	c.271dupA p.Arg91LysfsTer14	[20]
25	26/30	M	Sensorimotor neuropathy	Ataxia, anorexia, impaired short-term memory, confusion, cognitive decline, deep vein thrombosis, peripheral pulmonary embolism, depression	264	c.482G > A p.Arg161Gln	c.82-1G > A	[9]
26	26/33	F	Thrombotic microangiopathy	Nephrotic syndrome, renal failure, anemia	230	c.389A > G p.Tyr130Cys	c.271dupA p.Arg91LysfsTer14	[16]
27	28/29	M	Psychiatric symptoms	Cognitive decline, depression, euphoria, sleep disturbance, visual hallucinations, manic psychosis, lower limbs weakness, hyperreflexia, bilateral Babinski sign, visual decline, optic nerve atrophy, cortical atrophy	115.30	c.482G > A p.Arg161Gln	c.658_660del p.Ala221GlyfsTer7	[37]
28	28/29	M	Psychiatric symptoms	Euphoria, agitation, irritabilty, aggressiveness, mild memory impairment, bilateral paraplegia, pyramidal tract signs	75.7	c.482G > A p.Arg161Gln	c.656_658del p.Lys220ArgfsTer71	[32]
29	29/29	F	Low carnitine on daughter newborn screening	–	147	c.482G > A p.Arg161Gln	c.81 + 1G > A	[38]
30	29/29	M	Subclinical neuropathy	–	–	–	–	[14]
31	29/29	M	Ataxia	Neuropathy	102.8	c.482G > A p.Arg161Gln	c.656_658del p.Lys220ArgfsTer71	[8]
32	29/29	M	Psychiatric symptoms	Seizures	103.3	c.482G > A p.Arg161Gln	c.567dupT p.1190YfsTer13	[8]
33	30/32	M	Psychiatric symptoms	Euphoria, agitation, auditory and visual hallucinations, mild memory impairment, paraplegia, pyramidal signs, mild optic nerve atrophy	115.3	c.482G > A p.Arg161Gln	c.656_658del p.Lys220ArgfsTer71	[32]
34	31/33	M	Psychiatric symptoms	Insomnia, exaggerated expression, euphoria, increased irritability, thoughts of worthlessness, reduced vocal expression, social withdrawal, anorexia, lower limbs weakness, patellar tendons hyperreflexia, blurred vision, pigmentary retinopathy, optic nerve atrophy, cortical atrophy	65.0	c.482G > A p.Arg161Gln	c.658_660del p.Ala221GlyfsTer7	[37]
35	31/36	F	Psychiatric symptoms	Depression and psychosis requiring hospitalization, lower limbs weakness, legs paresthesia, lower limbs hemiplegia, thrombosis	57	c.482G > A p.Arg161Gln	c.271dupA p.Arg91LysfsTer14	[39]
36	32/34	F	Sensorimotor neuropathy	Ataxia, apathy, confusion, tetraparesis, anxiety, inability of self-care and communication, respiratory failure, deep vein thrombosis, white matter abnormalities, spinal cord involvement	53.3	c.347 T > C p.Leu116Pro	c.347 T > C p.Leu116Pro	[9]
37	32/Deceased	M	Neuropathy, lower limbs weakness	Numbness of extremities, ataxia, dysphagia, paraplegia, optic nerve atrophy, leukoencephalopathy, spinal cord atrophy	–	–	–	[26]
38	33/40	F	Recurrent venous thrombosis	Glomerulonephritis	288	c.365A > G p.His122Arg	c.271dupA p.Arg91LysfsTer14	[33]
39	35/35	M	Ataxia	Urinary incontinence, positive Romberg and Babinki signs	136.5	c.482G > A p.Arg161Gln	c.658_660del p.Ala221GlyfsTer7	[11]
40	38/39	M	Limb weakness, Ataxia	Neuropathy, scoliosis	67.1	c.80A > G p.Gln27Arg	c.609G > A p.Trp203Ter	[8]
41	38/42	M	Seizures	Cognitive decline, leukoencephalopathy	230	–	–	[27]
42	40/40	M	Cognitive decline	Cognitive decline, delirium, auditory hallucinations, ataxia, upper and lower limb rigidity, urinary incontinence, positive Babinski sign, cortical atrophy, cerebellar anomalies	57.2	c.482G > A p.Arg161Gln	c.1A > G p.Met1Val	[35]
43	41/42	M	Psychiatric symptoms	Depression, apraxia, ataxia, spasticity, myelopathy, pulmonary embolism, leukoencephalopathy, cervical/dorsal spinal cord atrophy	228	c.565C > A p.Arg189Ser	c.271dupA p.Arg91LysfsTer14	[33]
44	44/Deceased	M	Psychiatric symptoms, Cognitive decline	Social withdrawal, dysarthria, ataxia, optic nerve atrophy, deep veins thrombosis and pulmonary embolism, leukoencephalopathy	–	–	–	[26]
45	45/46	M	Depression	Cognitive decline, social withdrawal, insomnia, visual hallucinations, difficulty in following complex reasoning, ataxia, positive Babinski sign, seizures, renal failure, proteinuria, HUS, anemia, leukoencephalopathy, cortical atrophy	130	c.395_397del p.Arg132del	c.220delA p.Met74CysfsTer2	Present report

HUS, hemolytic uremic syndrome

Time between first symptoms and diagnosis ranged from 2 months to 21 years, with most (76%, 34/45) reported patients reaching a diagnosis within 2 years from symptoms onset.

The age for first symptoms at the onset of disease ranged from 18 to 45 years old. We subdivided the 45 patients into younger adults (i.e. 18–25 years old at disease onset; Group 1) and older adults (> 25 years old at disease onset; Group 2) and evaluated whether differences in the symptoms at onset could be highlighted (Table 2). Table 2 shows the distribution of the main onset symptom in the two age groups. Group 1 patients presented with neurological symptoms (encompassing neuropathy, lower limb weakness, paraplegia, ataxia and seizures) in 45% of cases (10 patients), followed by renal involvement at onset in 23% of cases (5 patients), neuropsychiatric presentation (term used when it was not possible to determine whether psychiatric or neurological symptoms presented first) in 18% of cases (4 patients) and open psychiatric onset in 9% of cases (2 patients). One patient (5%) had a pulmonary embolism as a first presenting symptom. Group 2 patients presented with open psychiatric symptoms in 39% of cases (9 patients) and neurological symptoms in 39% of cases (9 patients). One patient presented with isolated ocular involvement (4.3%) and one (4.3%) with thromboembolic disease (recurrent venous thrombosis). Furthermore, the two aforementioned asymptomatic patients were both older than 25 years of age when their biochemical evaluation was performed (9%). The Chi-square test shows statistically significant differences among symptoms at onset in the two age groups (Table 2).

**Table 2:** Symptoms at onset: comparison between group 1 (18–25 yo) and group 2 (26–45 yo)

Symptoms at onset	Group 1 (n.: 22)		Group 2 (n.: 23)	
	n	%	n	%
Neurological symptoms	10	45	9	39
Renal involvement	5	23	1	4.3
Neuropsychiatric symptoms	4	18	0	0
Isolated psychiatric symptoms	2	9	9	39
Thromboembolic disease	1	5	1	4.3
Ocular symptoms	0	0	1	4.3
Asymptomatic	0	0	2	9

Chi-square value	14,092
Degrees of freedom	7
*P* value	0.0286
Rows × columns	2 × 8

**Fig. 6:**
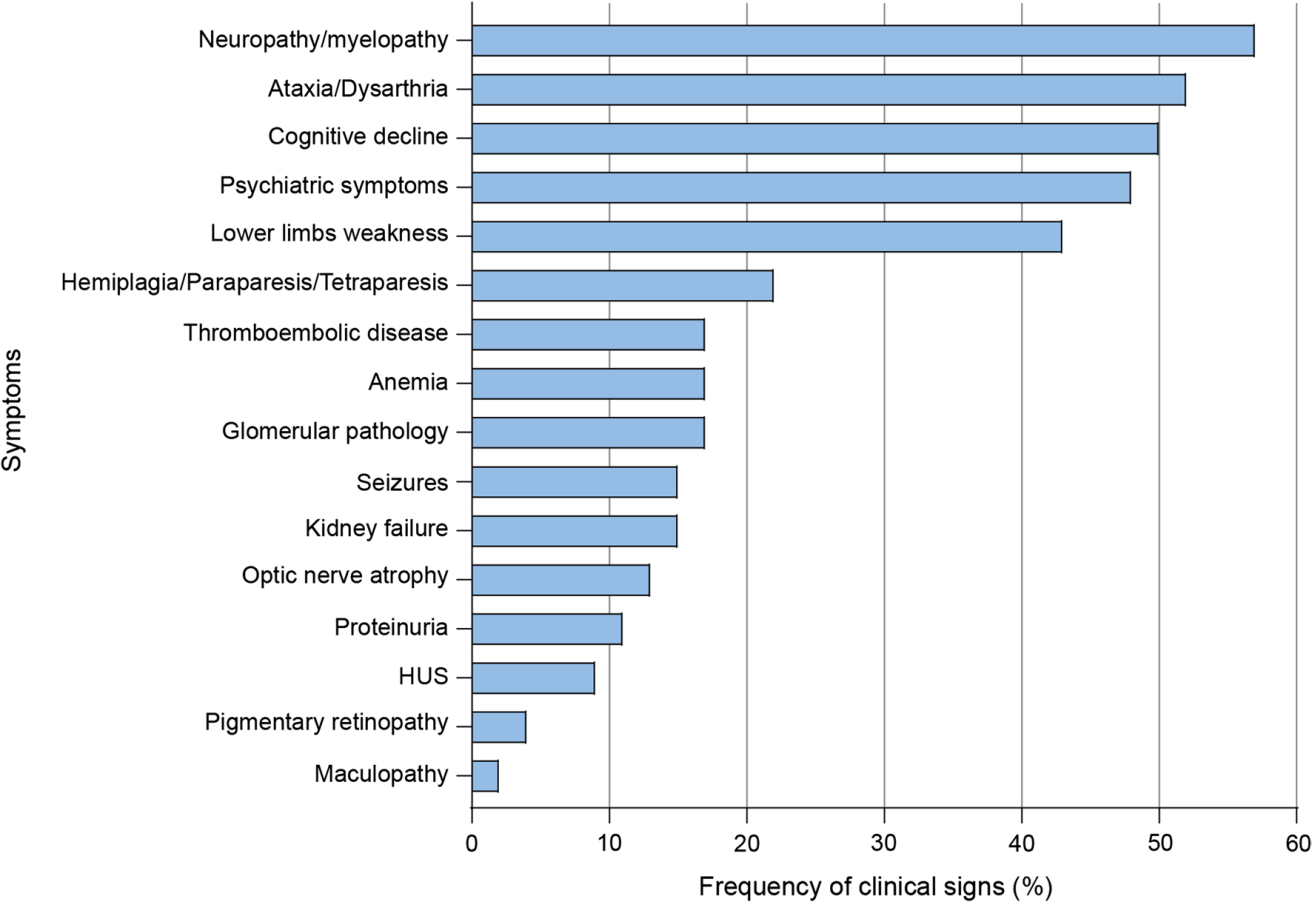
Frequency of clinical signs and symptoms in adult-onset patients. Incidence of overall symptoms with disease progression: the X-axis shows the percentage of symptoms, while Y-axis lists the symptoms of CblC disease

Besides the first symptoms at onset, the disease progressed in most patients. Figure 6 shows the incidence of overall symptoms: on the Y-axis of the graph are the symptoms, and on the X-axis the percentage of such symptoms per age range is displayed.

Peripheral nervous system (neuropathy/myelopathy) involvement was the most frequent associated symptom overall (57%). Characteristic of the disease is a sensory-motor axonal neuropathy predominantly in the lower limbs. Lower limb weakness is one of the most common clinical manifestations of the neuropathy and was a presenting symptom in 7 patients (16%) and was reported overall in 20 patients over the course of the disease (43%). Spinal cord progressive motor involvement even manifested as hemiplegia, paraparesis or tetraparesis in 22% of patients later in the course of disease.

Cognitive decline, manifesting as a decline in school and work performance, was a common associated symptom, present in 50% of adult-onset cases. Oftentimes cognitive decline was not recognized as an organic manifestation, as it presented before any anomaly could be seen on brain imaging, and was then believed to be psychiatric. Pyramidal tract involvement manifesting as ataxia or dysarthria was present in 52% of cases. Psychiatric symptoms were overall present in 48% of the adult-onset patients. Recurrent psychiatric symptoms ranged from visual and auditory hallucinations, euphoria, psychosis, sleep disturbances, irritable behavior, delirium to social withdrawal and depressive symptoms. Seizures were overall present in 15% of patients during disease progression.

Kidney involvement of CblC deficiency consist of variable degree proteinuria (also up to the nephrotic range), hypertension (also malignant hypertension), chronic renal failure (from mild to severe) and hemolytic uremic syndrome. Glomerular disease (17% overall) and kidney failure (15% overall) were relatively common in both Group 1 and Group 2, despite being a presenting symptom mainly in younger patients (Table 2). Proteinuria was reported in 11% of adult patients and atypical hemolytic uremic syndrome in 9%.

Optic nerve atrophy was the most common visual involvement (13%). Pigmentary retinopathy and maculopathy were instead reported in 4% (2 patients) and 2% (1 patients) of adult patients, respectively. Normocytic anemia was mentioned in 17% of patients.

55.6% (25/45) of patients showed some abnormality on either brain or spinal cord imaging. Cortical atrophy was the most common imaging abnormality (30% of all adult patients), spinal cord degeneration was instead found in 28% of adult patients and 20% of patients had some white matter anomaly in brain imaging. Imaging anomalies were not present at disease onset in most cases (Additional file 1: Table S1).

Homocysteine at diagnosis had an average value of 137.4 μmol/l (SD 70), with a minimum value of 27.9 μmol/l to a maximum value of 288 μmol/l. The values of methylmalonic acid and methionine were not consistently present, and varied depending on variable creatinine values, not always reported (Additional file 1: Table S1 for further details).

Response to treatment was described as optimal in all cases both from a clinical and biochemical point of view. Homocysteine values after treatment were not always present, but all authors described marked improvement of all parameters within a few weeks of treatment initiation (Additional file 1: Table S1 for further details). The mainstay of therapy was always parenteral hydroxycobalamin with doses ranging from 1 mg/day to 0.5–1 mg/week, which was variably combined with folic acid, betaine and carnitine (see Additional file 1: Table S1 for further details).

**Fig. 7:**
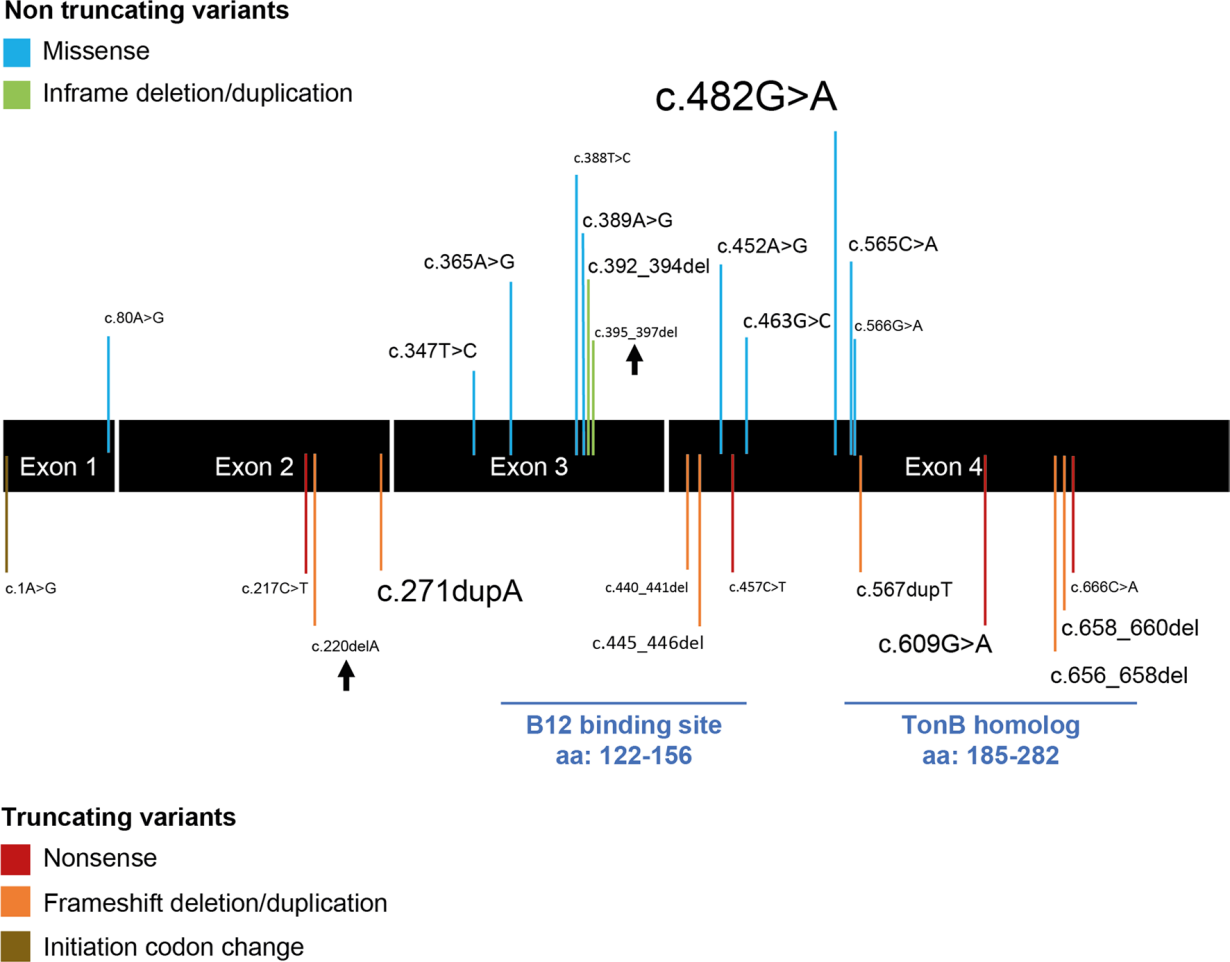
Patients’ cohort genotype. *MMACHC* gene structure and coding pathogenic variants reported in association with adult-onset CblC disease. The non-truncating variants are shown in the upper part of the figure, while the truncating ones are showed in the lower part. The three splicing variants c.81 + 1G > A, c.82-1G > A and c.82-12_82-9delTTTC are not represented in the figure, but they all mapped into the intron 1 of the gene. The size of character of the variants is proportional to the frequency of their occurrence in the cohort. Known protein domains are indicated in light blue. The two new variants found in our patients are highlighted by arrows

As far as the patients’ genotype is concerned, 27 different variants have been associated to the adult-onset phenotype (Fig. 7). Four patients (patient 8, 9, 17 and 36) showed a homozygous pathogenic variant (Table 1). All the remaining cases presented compound heterozygous variants. Specifically, they presented with a combination of a truncating variant (nonsense, frameshift, initiation codon change or splicing) and non-truncating variant (missense, inframe deletion/duplication). The most frequently occurring variant in the cohort is c.482G > A, present in 21 patients, followed by c.271dupA (9 patients), c.609G > A (6 patients), c.656_658del (4 patients), c.658_660del (4 patients) and c.452A > G (homozygous in two patients). The c.365A > G, c.445_446del, c.347 T > C variants occurred three times, while variants c.389A > G, c.392_394del, c.565C > A, c.567dupT were present twice. The remaining 14 variants were reported in single patients (Fig. 7). No genotype was available for five patients, whose reports occurred prior to the discovery of the gene associated with the disease (patient 10, 30, 37, 41 and 44).

### Renal biopsy characteristics in CblC deficiency

Although renal involvement is more frequent in early onset disease, no significant differences are described between the clinical nephrological pictures of the infancy and adult forms of CblC deficiency.

Kidney biopsies usually show the typical lesions of acute and chronic thrombotic microangiopathy (TMA) [15, 16]. Vessels present intravascular thrombi, fibrous endarteritis and hyalinization. Glomeruli present thrombi, glomerular ischemia and remodeling of the glomerular basement membrane (GBM), visible as duplication and/or vacuolar aspect of the GBM and/or double contours.

While there are not significant histological differences in the aspect of microangiopathic nephropathy between childhood and adult forms of CblC deficiency, it is interesting to point out that in a comparison of 7 patients with CblC TMA with 16 matched controls with CblC-independent TMA the Authors found a more vacuolated appearance of the GBM (86% vs 7%) and a more frequent and abundant glomerular IgM deposition among patients with CblC deficiency [16].

Immunofluorescence study of renal biopsies mostly did not reveal immune deposits in early onset CblC deficiency, while cases with presence of IgM, C3 and less frequently IgA and C1q are described in older patients [16–18]. Such a finding might be misleading, as happened for a 16-year-old pediatric case with a renal biopsy similar to our patient, for which atypical glomerulopathy was suspected [19]. Our patient presented a strong immunofluorescence positivity for C1q and IgG, together with double contouring of the GBM and deposits structured in a microtubular fashion, mimicking a cryoglobulinemic glomerulonephritis. Similarly to what was described by *Lemoine *et al. [16], our patient showed chronic thrombotic microangiopathic features with glomerular double contours and occasional intravascular thrombi, but, differently from them, we reported associated dominant IgG and C1q deposition and we did not find significant vacuolated appearance of the GBM with electron microscopy, but a more prominent “multilayering” aspect.

## Discussion

We here report the first comprehensive description of CblC deficiency in adults, encompassing data from 45 patients. The disease has been thoroughly studied and is commonly recognized in the pediatric population, while there is a lack of awareness among adult patients’ physicians.

In particular, since the onset of symptoms can vary widely, the first specialist seeing an adult patient affected by CblC deficiency can be either a psychiatrist, a neurologist, a nephrologist, an ophthalmologist or an internist, and it is therefore of paramount importance that all these figures are aware of the disease and sensible to other signs and symptoms, not inherently belonging to their area, but whose presence could orientate towards the correct diagnosis.

As opposed to pediatric patients, the onset of the disease is often very insidious in adults, with psychiatric symptoms and slow cognitive decline, without the characteristic brain imaging anomalies from the very beginning. Mild psychiatric symptoms such as introversion and paranoia can be neglected for a long time, as shown by our case report and previous series describing patients with neuropsychiatric onset [11].

A slow and steady decline of renal function may come altogether, as described in our case report. While anemia was reported in 18% of patients from the adult-onset cohort, it is never macrocytic and this may drag the attention away from a deficit of vitamin B12. Furthermore, due to the coexistence of psychiatric symptoms, the signs of neurological decline and neuropathy may be noticed later due to lower reliability and awareness of the patient.

The full spectrum of adult-onset symptoms includes neuropathy, ataxia, dysarthria, cognitive decline, psychiatric symptoms (ranging from depression to visual and auditory hallucinations), lower limb weakness, hemiplegia, paraparesis, tetraparesis, epileptic seizures, glomerular pathology with proteinuria, decline of renal function, thromboembolic disease, atypical hemolytic-uremic syndrome, optic nerve atrophy, and, in rare cases, pigmentary retinopathy and maculopathy (Fig. 6). Pulmonary hypertension, typically present in children with the disease, has never been described in adult patients. Visual impairment, commonly present in the early onset form, is only rarely observed in adults. However, a single case report of a woman with isolated bull’s eye maculopathy demonstrated that the visual symptoms may be the only presenting complaint [20].

Despite being commonly mentioned, psychiatric symptoms were often not described in details. The most recurrent psychiatric symptoms reported were visual and auditory hallucinations, euphoria, psychosis, sleep disturbances, irritable behavior, delirium, social withdrawal and depressive symptoms.

As far as the renal involvement is concerned, it is important to remind that microangiopathic nephropathy can be present in the complete absence of the laboratory alterations typical of TMA, as in the case of our patient [21]. It is therefore important to suspect the disease when the typical histological features of TMA are seen in a renal biopsy performed for idiopathic kidney failure and that homocysteine dosage is included in the diagnostic flow-chart of TMA nephropathy. Furthermore, since in chronic renal failure total homocysteine levels can exceed the upper-normal limit by 2–5 fold [22], dosage of MMA and Met can possibly exclude any vitamin B12 metabolism alteration. It should also be noted that in our patient, as described in previous cases [17], eculizumab was absolutely ineffective in resolving the HUS. It is therefore mandatory to dose homocysteine in patients with eculizumab resistant HUS, as already stated in the guidelines for HUS management [23].

Although presenting symptoms may vary widely, plasma homocysteine levels are a reliable and easy way to reach a diagnosis. Whenever an intracellular cobalamin metabolism defect is clinically suspected, plasma homocysteine must be asked for. When hyperhomocysteinemia is found [especially when > 50 μmol/l [2]], methionine plasma levels and methylmalonic acid levels in urine and/or plasma must be requested in order to discriminate among the different forms of remethylation disorders [reviewed in [2]]. In CblC defects methylmalonic acid in plasma and urine is elevated [plasma MMA > 100 μmol/l, and urine’s MMA > 500 μmol/l [2]] as a consequence of the impaired conversion of methylmalonic into succinic acid, mediated by the cofactor adenosylcobalamin. At the same time plasma methionine levels are low [Met < 13.4 μmol/L [4]], as a consequence of the impaired homocysteine remethylation to methionine which is normally mediated by the cofactor methylcobalamin, no longer present (Fig. 1). In the adult cohort analyzed, the average value of homocysteine at symptoms onset was 172 μmol/L, with values reported as low as 27.9 [12] and as high as 273.3 μmol/L [8]. It was not possible to estimate the average values of MMA and Met, as these were not always present or, in the case of urinary MMA, the value of the patient creatinine was not available.

As far as molecular findings are concerned, it is remarkable to notice that the adult-onset cases are mostly characterized by compound heterozygosity with a strongly deleterious truncating variant (nonsense, frameshift, initiation codon change or splicing) and a milder one (missense, inframe deletion/duplication). A residual function of the protein is thus maintained, with consequences of the molecular anomaly seen only later on in life. The predominant variant in the adult-onset cohort is c.482G > A, a variant already associated with a milder clinical and biochemical phenotype (Fig. 7) [24]. The second most prevalent variant in the cohort is c.271dupA, the most common variant among all individuals of European ancestry affected by CblC [9]. In the adult onset though, the c.271dupA variant is always present as compound heterozygous with a non-truncating one. c.609G > A is the third most common variant in our cohort and the most common among Chinese individuals affected by CblC disease, irrespective of the age group [25]. From a molecular genetics point of view, there seems to exist a genotype–phenotype correlation: the older adult cases tend to have two different pathogenic variants, one of which has a milder effect on the protein that seems to retain a residual function. In the early-onset cases on the other hand, the protein is virtually nonfunctional, leading to early symptoms, some of which cannot be treated despite early therapy following an altered newborn screening. This notwithstanding, even among adult onset cases, there is a very variable clinical presentation associated to the very same pathogenic variants within members from the same family, as shown by the siblings described by Gold et al. [14] (patient 30 Table 1 and sister with onset of symptoms at age 12), Powers et al. [26] (patients 37 and 44 Table 1), and Boxer et al. [27] (patient 41 Table 1 and sister with onset of symptoms at age 6). As in other monogenic disorders, the reason for this variability likely roots back to other environmental factors, including diet, and the rest of the genetic background acting as a phenotype modifier [11].

From a diagnostic standpoint, once homocysteine, methionine and MMA levels have been measured, we believe that it would be important to analyze the patients with a suspected intracellular cobalamin disorder with NGS. In particular, since it is common practice to analyze a panel of complement genes when atypical hemolytic uremic syndrome is present, we suggest that such panels are integrated with genes belonging to the intracellular cobalamin metabolism and folate pathway, specifically: *ABCD4* (CblJ disease, OMIM #614857), *HCFC1* (CblX disease, OMIM #309541), *LMBRD1* (CblF disease, OMIM #277380), *MMACHC* (CblC disease, OMIM #277400), *MMADHC* (CblD disease, OMIM #277410), *MTHFR* (Homocystinuria due to MTHFR deficiency, OMIM #236250), *MTRR* (CblE disease, OMIM #236270), *MTR* (CblG disease, OMIM # 250940), *MTHFD1* (Combined immunodeficiency and megaloblastic anemia with or without hyperhomocysteinemia, OMIM #617780) and *PRDX1* (associated to CblC disease, OMIM #277400). The role of most of the proteins encoded by these genes is shown in Fig. 1. Such a practice would have spared our patients months of delay in the diagnosis.

CblC disease is one of the secondary targets in newborn screening, detected through propionylcarnitine (C3). Extended neonatal screening including CblC disease has been uniformly applied in Italy since 2017 [28]. As a consequence, adult patients nowadays did not benefit from it as neonates and it is therefore important to be aware of the disease and its subtle onset in this population. Nonetheless, it has been previously reported that heterozygous carriers of milder variants may not be identified in newborn screening due to absence of biochemical anomalies at birth [24].

In pediatric patients, especially those with the early onset form of the disease, early treatment is overall effective on disease progression. However, neurodevelopmental delay and ophthalmological anomalies tend to progress despite treatment. This is most likely due to brain and optic damage occurring already during fetal life due to a complete absence of MMACHC protein. On the other hand, residual protein function in adult-onset cases, allows for complete symptoms resolution, further highlighting the value of a timely diagnosis. As demonstrated by the brothers described by *Bodamer *et al. in 2001 [12], despite milder disease and later onset of symptoms in adult CblC disease, lack of diagnosis inevitably leads to the patients’ death.

The mainstay of therapy is parenteral hydroxycobalamin, combined with betaine (250 mg/kg/ day) and folic acid (1 mg/day). Hydroxycobalamin dosages are quite variable in clinical practice, ranging from 1 mg/die to 10 mg/day [29, 30].

Due to the subtle insidious presentation of the disease, the diagnosis has likely been missed or delayed in many instances. We believe that the relatively low number of patients with adult onset CblC disease may be due to lack of awareness among adult patients’ physicians and we hope that increased knowledge about disease course in adults and measurement of plasma homocysteine could improve the diagnostic rate with marked improvement of the prognosis.

A limitation of our retrospective analysis of the published adult onset CblC cases is that oftentimes the accuracy of the reported symptoms and the disease onset depended on the expertise of the authors. An emblematic example is the case report from *Philipponnet and colleagues* where the focus was the renal phenotype, while it was only mentioned that the patient had a “neurological impairment”, whose characteristics are unknown [31]. We believe that further knowledge and insight on the disorder is going to pave the way for more accurate recognition and description of all the associated symptoms and better care for the patients.

## Conclusion

CblC deficiency is an inherited cobalamin metabolism disorder that may present in adult patients, as shown by our case report and the systematic review of the literature. Due to the variability of clinical presentation and age of onset, it may represent a diagnostic conundrum for adult medicine physicians. Many symptoms are indeed variably present and the risk is, as happened for our patient, that they are considered as separate entities and each is treated independently by a different medical specialist.

In order to avoid misdiagnosis we therefore suggest that adult onset CblC deficiency is acknowledged as a separate entity from pediatric late onset cases, and that the disease is considered in the differential diagnosis in adult patients with aHUS and/or slow unexplained decline in renal function and/or idiopathic peripheral neuropathies, spinal cord degenerations, ataxias and/or recurrent thrombosis and/or visual field defects, maculopathy and optic disc atrophy. Furthermore, as we recognize that subtle psychiatric onset in absence of other organic anomalies is a very common isolated initial presentation in adults, we suggest that a high degree of suspicion is maintained by psychiatrist with regards to intracellular vitamin B12 metabolism disorders.

Plasma homocysteine is a key biochemical indicator for remethylation disorders and should be used as a screening for differential diagnosis of the all the above-mentioned conditions. Homocysteine measurement is cheap and widely available in most centers following adult patients. We advocate that it should be performed beyond thrombophilia screening and kept in consideration by neurologists, nephrologists, ophthalmologists, internal medicine doctors and psychiatrist alike.

It must furthermore be borne in mind that vitamin B12 and folate serum levels will always be normal in these patients. They do have a functional deficit of cobalamin, due to impaired intracellular processing of the vitamin, but the plasma levels of cobalamin are normal and macrocytic anemia is not typically present.

In contrast with its early onset form, therapy is effective on all symptoms’ progression, underscoring the importance of recognizing this rare, but treatable disorder in adulthood.

To further aid diagnosis of the disorder, it is important that genes belonging to the intracellular cobalamin pathway are included within gene panels routinely performed for aHUS and chronic kidney disorders.

We believe that with lower costs of molecular genetic testing and increased clinical awareness, CblC deficiency will be increasingly diagnosed in adults, saving the patients a long and gruesome diagnostic odyssey.

## Methods

### Next-Generation sequencing and data analysis

Genomic DNA was extracted from peripheral blood using “DNeasy” Kit (Qiagen) and checked for integrity by gel electrophoresis (E-gel system, Thermo fisher). Next-generation sequencing was performed based on a clinical exome sequencing including 6,700 genes (Illumina), raw data aligned to the hg19 human genome reference using the DRAGEN Enrichment v.3.8.4 tool (Illumina) and data analysis focused on a panel of 9 genes related to vitamin B12 intracellular metabolism (*ABCD4; HCFC1; LMBRD1; MMACHC; MMADHC; MTRR; MTR; MTHFD1; MTHFR*). Variant annotation and curation were performed using Variant interpreter (Illumina), Alamut (Sophia Genetics) and different databases, including Varsome, dbSNP, Mastermind, UniProt.

### Sanger sequencing

Sanger sequencing was used to validate the identified variants in our patient and for family segregation. PCR reactions were carried out in 30 μl volumes containing 25 ng of genomic DNA, 0.7 μM of each primer, 1.7 mM MgCl2, Go Taq buffer 1X, 0.2 mM dNTP, and 1,25 units of GoTaq Hot Start Polymerase (Promega, Madison, Wisconsin, US). The PCR products, after purification by QIAquick PCR Purification kit (Qiagen, Hilden, Germany), were sequenced using BigDye terminator cycle sequencer system v3.1 (Applied Biosystems, Foster City, California, US). Sequence analysis was performed on ABI Prism 3100xl Genetic analyzer (Applied Biosystems). The base-called sequences were aligned on the reference sequence to *MMACHC* gene (NG_013378.1) using Chromas Lite software (http://technelysium.com.au/) and all chromatograms were confirmed visually. For amplification and sequencing we used 4 primers designed with Primer-BLAST (https://www.ncbi.nlm.nih.gov/tools/primer-blast/).

### Systematic review of the literature

The PubMed database was searched using the terms “cobalamin; CblC; MMACHC; combined methylmalonic aciduria homocystinuria; MMA; homocysteine”. The terms were combined with “late onset” and “adult onset”. Reviews, case reports and case series published before August 2021 were considered. Furthermore, references listed in the papers retrieved were also screened for relevant cases. All cases with first symptom at onset ≥ 18 years old were included. Data on clinical symptoms at onset and during disease development, therapy, biochemical and molecular data were collected, whenever included in the original articles. Only articles that were written in English were taken into consideration.

### Statistical analysis

The Chi-square test was used to compare Group 1 and Group 2 in terms of symptoms and relevant values reported in Table 2. Statistical analysis was performed using the GraphPad Prim software.

## Supplementary Information

**Additional file 1**. Complete clinical, biochemical and molecular data on the 45 patients' cohort.

## Data Availability

The dataset supporting the conclusions of this article is included within the article and its additional file.

## Abbreviations

aHUS: Atypical hemolytic uremic syndrome
CblC: Cobalamin C
GBM: Glomerular basement membrane
Hcy: Homocysteine
im: Intramuscular
LDH: Lactate Dehydrogenase
Met: Methionine
MMA: Methylmalonic acid
MMACHC: Methylmalonic Aciduria And Homocystinuria Type C Protein
NGS: Next Generations Sequencing
PRES: Posterior reversible encephalopathy syndrome
sCr: Serum Creatinine
SD: Standard Deviation
TMA: Thrombotic microangiopathy
